# Supplementary material for: Auditory cortical representation of music favours the perceived beat
Source: R Soc Open Sci. 2020 Mar 25;7(3):191194. doi: 10.1098/rsos.191194 (PMC7137933; doi:10.1098/rsos.191194)
Supplement: Supplementary Information [file RSOS191194supp1.docx]

**Electronic Supplementary Material**

**Supplementary Table 1. Summary of musical excerpts.** Tempo in beats per minute (BPM) based on consensus inter-tap interval across the 40 human annotators.

| **Excerpt** | **Title** | **Artist** | **Genre** | **BPM** |
| --- | --- | --- | --- | --- |
| 1 | You're The First, The Last, My Everything | Barry White | R&B/Soul | 129 |
| 2 | A New England | Billy Bragg | Alternative/Indie/Folk | 82 |
| 3 | El Contrapunto | Los Mensajeros De Las Libertad | Latin/Folk/World | 152 |
| 4 | Green Eyes | Erykah Badu | Contemporary Soul | 42 |
| 5 | Passe & Medio Den Iersten Gaillar | Josquin Des Prez | Classical | 68 |
| 6 | Wo Ai Ni | Thai China Dolls | Pop | 82 |
| 7 | Le Sacre du Printemps | Igor Stravinsky | Classical | 57 |
| 8 | Flim | Aphex Twin | Electronica | 148 |
| 9 | Hurricane | Bob Dylan | Classic Rock | 127 |
| 10 | Vespro della beata Vergine | Claudio Monteverdi | Classical/Opera | 62 |
| 11 | Komm nach Tirol | Zillertaler Schürzenjager | Folk | 141 |
| 12 | Matthäus-Passion, BWV 244 | Johann Sebastian Bach | Classical | 54 |
| 13 | Kalasnjikov | Goran Bregović | Rock/Jazz | 181 |
| 14 | Not Gonna Get Us | t.A.T.u. | Pop | 130 |
| 15 | Le Bruit Du Frigo | Mano Negra | Rock/Folk | 63 |
| 16 | La Carpinese (Tarantella) | Lucilla Galeazzi | Classical | 92 |
| 17 | The Piano Has Been Drinking [Not Me] | Tom Waits | Alternative/Indie | 46 |
| 18 | Exit Music (For A Film) | Radiohead | Alternative Rock | 62 |
| 19 | Possessed To Skate | Suicidal Tendencies | Metal | 190 |
| 20 | El Gato Lopez | Ska-P | Ska | 222 |


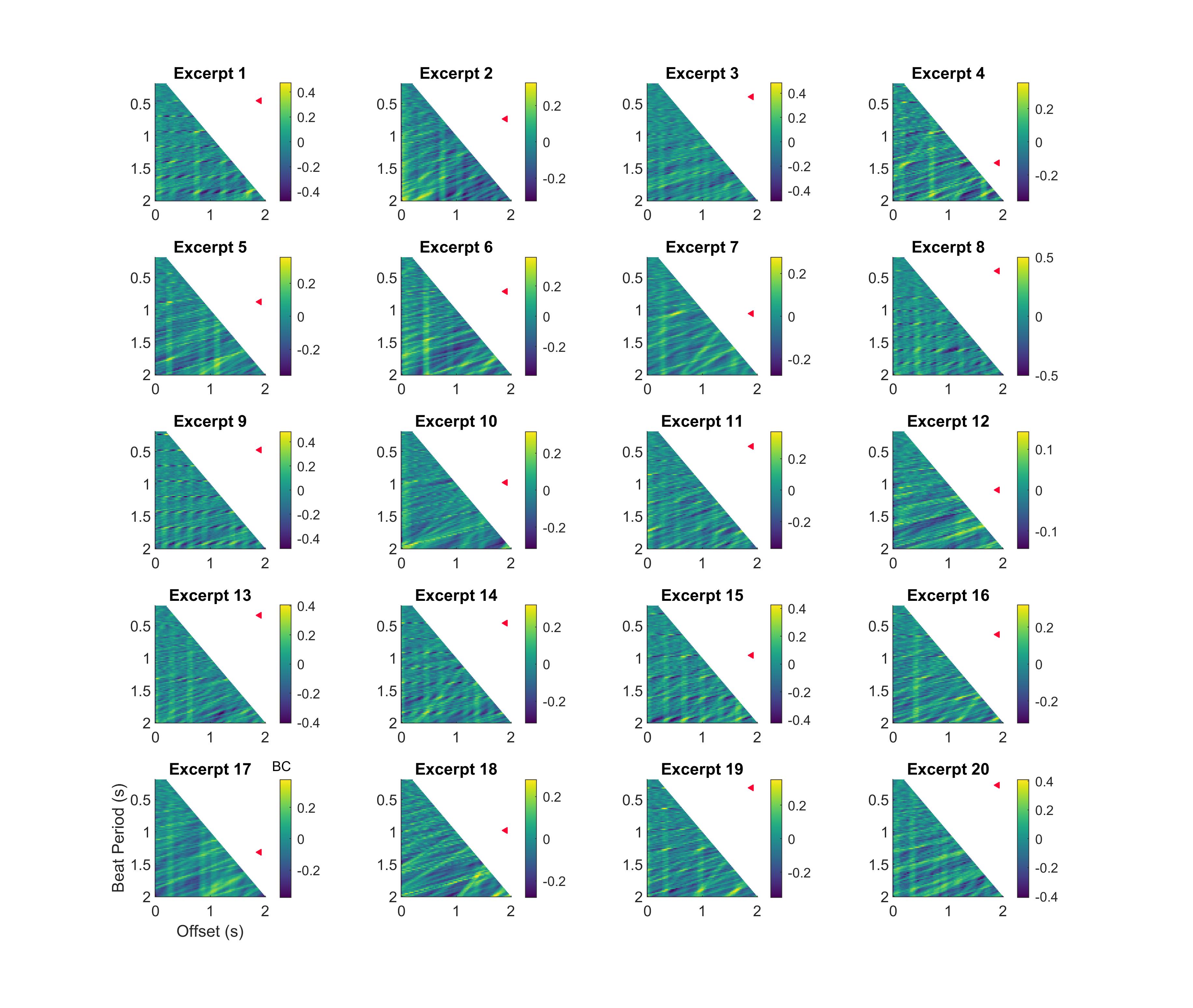


**Supplementary Fig. S1. Hypothetical beat contrasts in auditory cortex for possible beat interpretations.** Each panel is one excerpt, as labelled in the MIREX 2006 database. Colours show BC values for beat period (y-axis) and beat phase (x-axis) combinations between 200 ms and 2 s (or tap rates of 5 Hz down to 0.5 Hz) in 0.02 s increments. Red triangles mark the row corresponding to that song’s consensus beat period. See Fig. 3A in the main text.


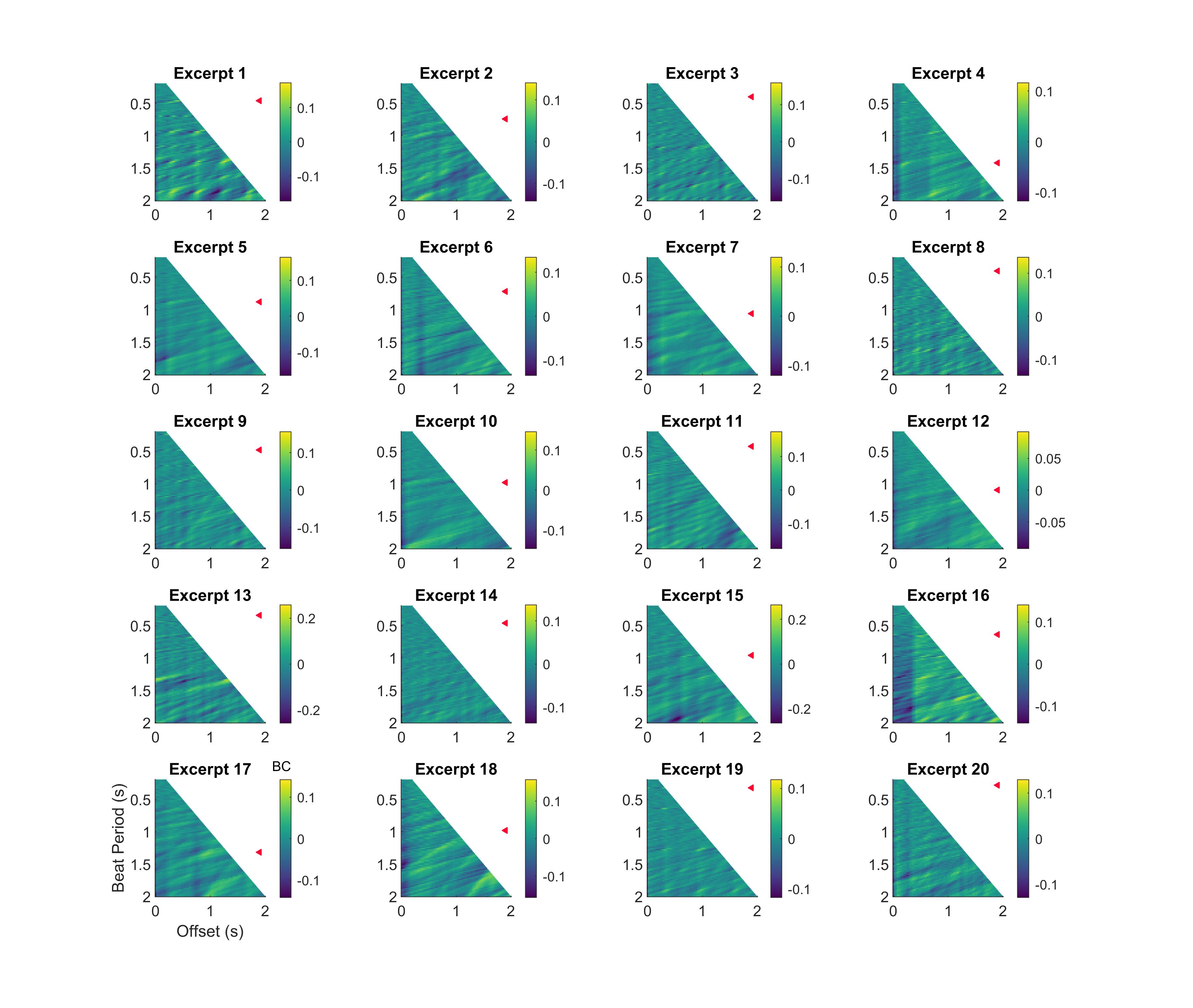


**Supplementary Fig. S2. Hypothetical beat contrasts for AN model for possible beat interpretations.** Each panel is one musical excerpt, as labelled in the MIREX 2006 database. Colours show BC values for beat period (y-axis) and beat phase (x-axis) combinations between 200 ms and 2 s (or tap rates of 5 Hz down to 0.5 Hz) in 0.02 s increments. Red triangles mark the row corresponding to that song’s consensus beat period. Note the difference in range of BCs compared to cortical data in Fig. S1. See Fig. 3D in the main text.


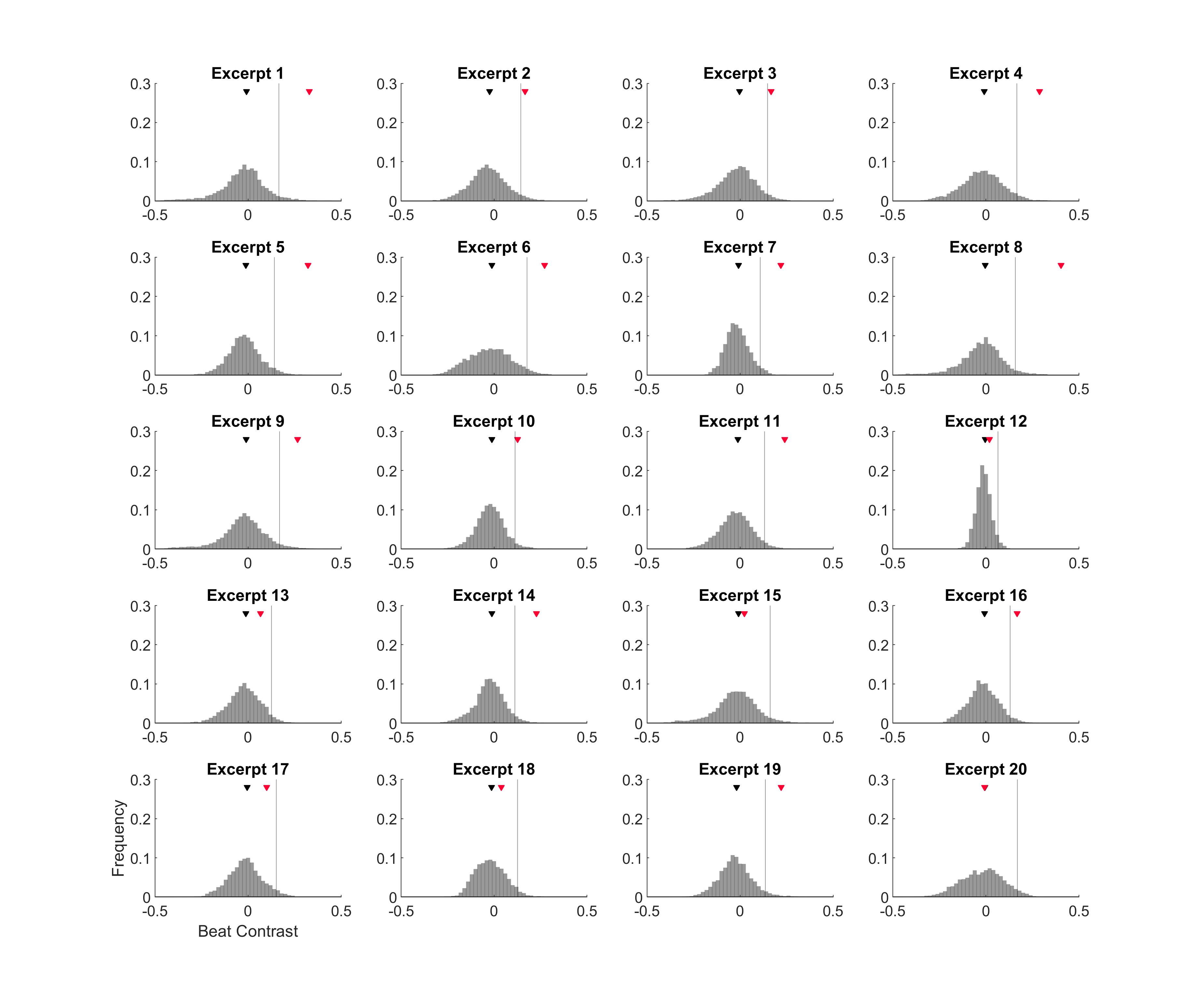


**Supplementary Fig. S3. Hypothetical BC distributions in the auditory cortex for each song.** Each panel is one musical excerpt, as labelled in the MIREX 2006 database. Histograms show BC values at all sampled hypothetical beat period and beat phase combinations for a given piece of music. The black triangle shows the median of this distribution, and the red triangle shows the song’s consensus BC value, and vertical gray lines mark the 95^th^ percentile. The consensus BC is high relative to the underlying distribution of BCs for most songs. See Fig. 3B in the main text.


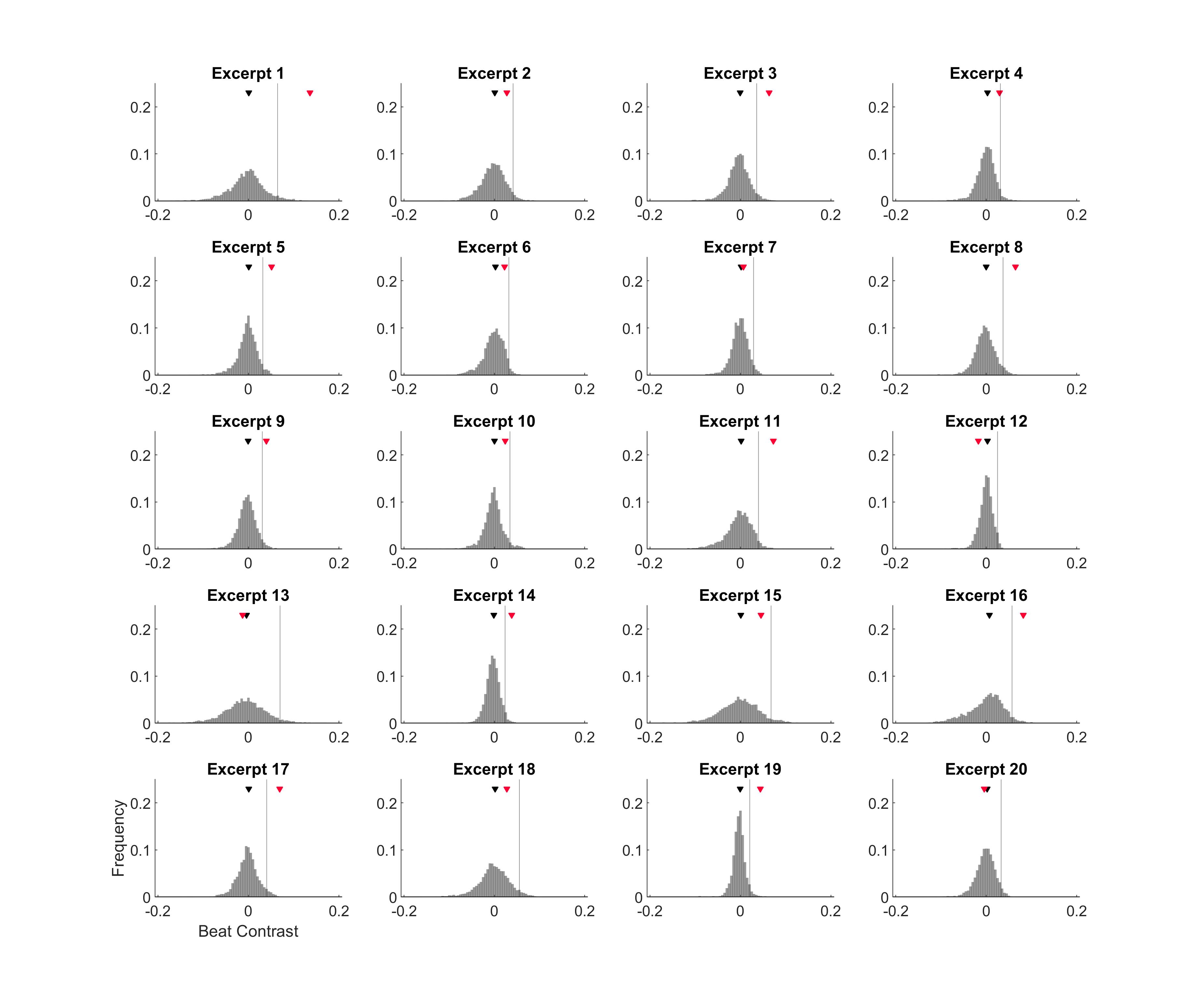


**Supplementary Fig. S4. Hypothetical BC distributions based on AN model for each song.** Each panel is one musical excerpt, as labelled in the MIREX 2006 database. Histograms show BC values at all sampled hypothetical beat period and beat phase combinations for a given piece of music. The black triangle shows the median of this distribution, and the red triangle shows the song’s consensus BC value, and vertical gray lines mark the 95^th^ percentile. See Fig. 3E in the main text.


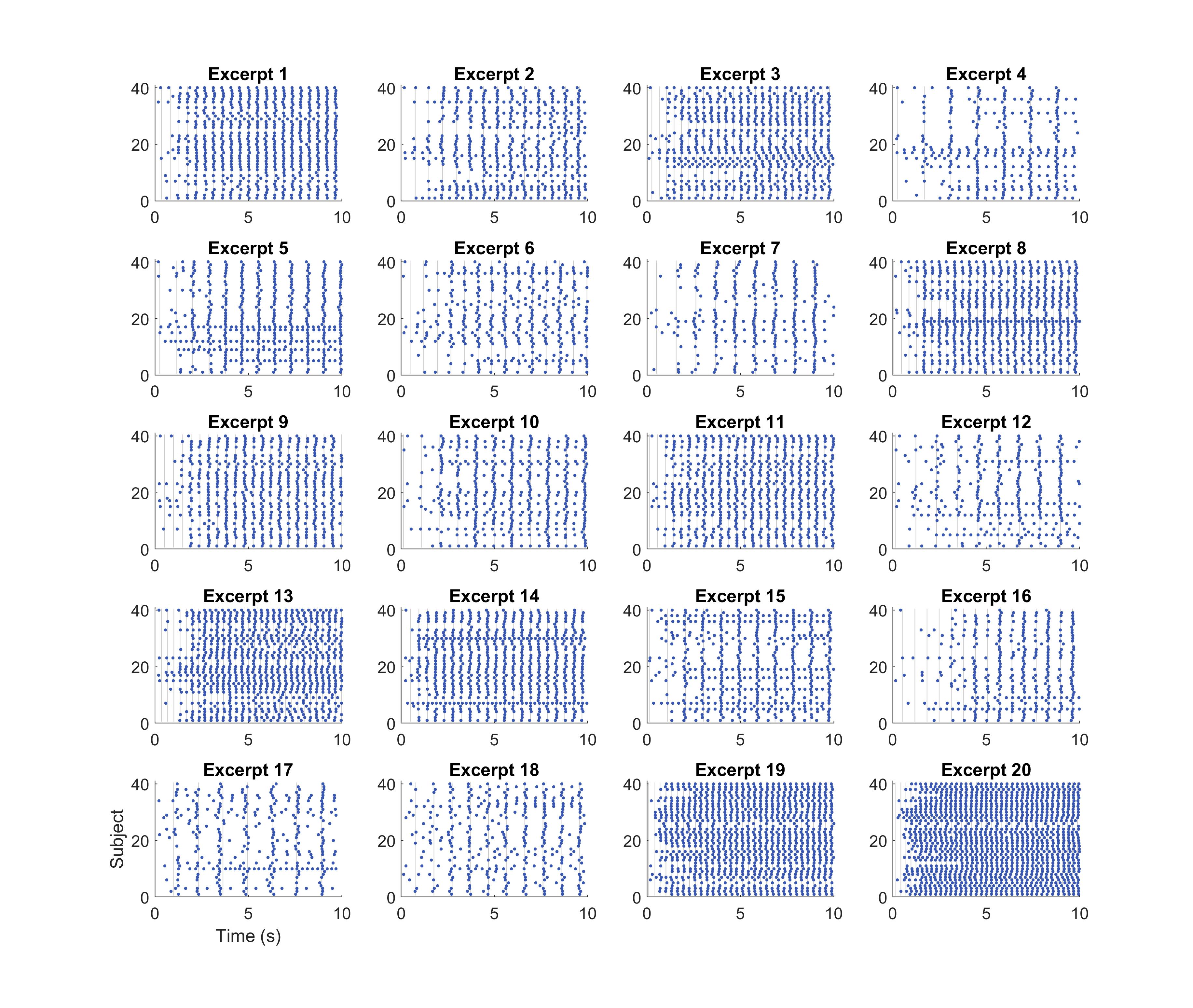


**Supplementary Fig. S5. Tap rasters for all songs.** Each panel is one musical excerpt, as labelled in the MIREX 2006 database. Each dot is one tap, each row is one subject and the position of taps along the x-axis represents when that subject tapped.


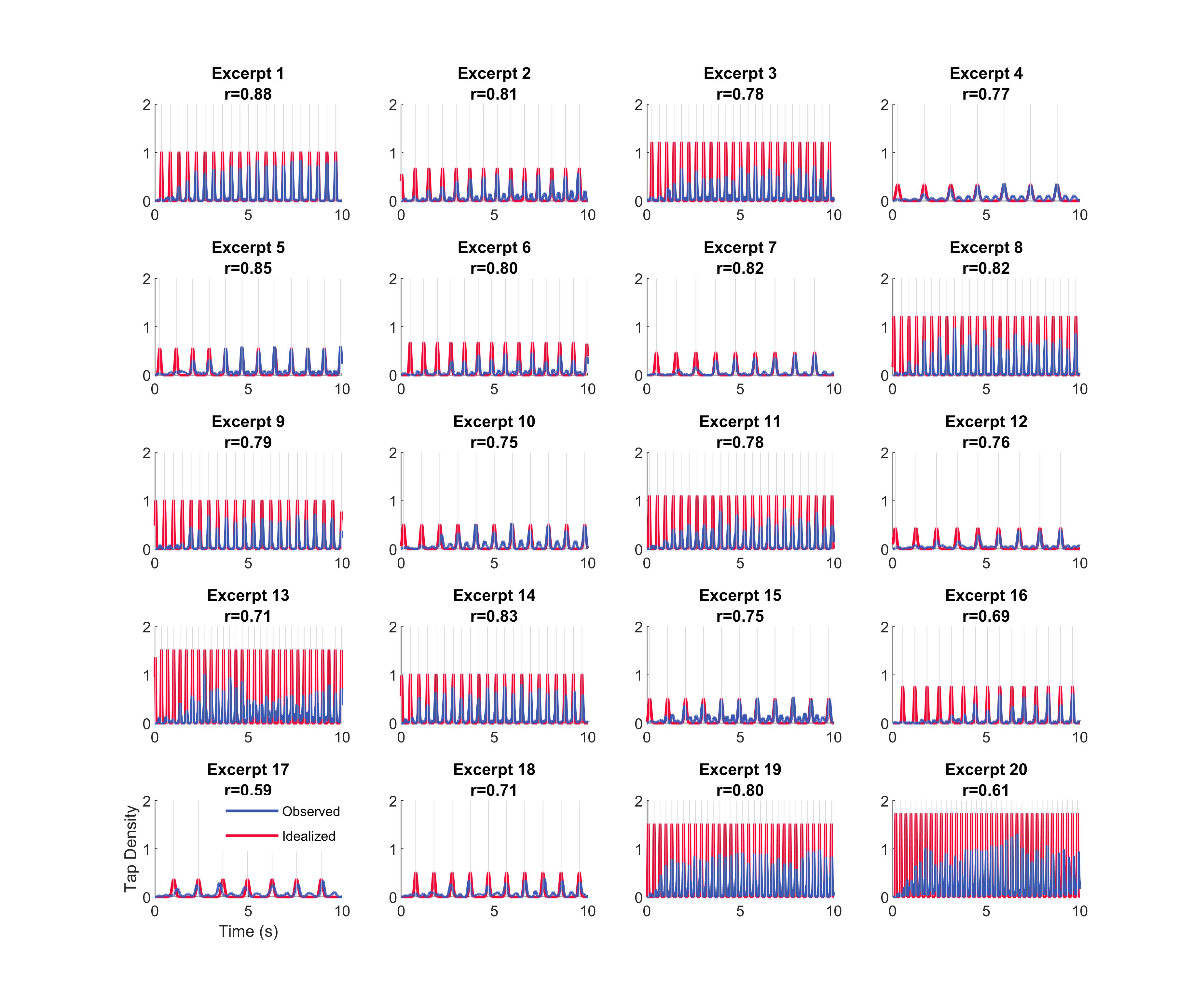


**Supplementary Fig. S6. Observed and idealised tap density estimates.** Each panel is one musical excerpt, as labelled in the MIREX 2006 database. Tap density estimates are based on tap times pooled across subjects, binned with 2 ms bins, and smoothed with a Gaussian kernel with a standard deviation of 5% of the consensus beat period (blue). Shown in red is a tap density estimate of the “ideal” tap histogram (with realistic motor error) that would have been obtained if all subjects had tapped on every consensus beat (see *Methods*). The correlation coefficient (r) between real and idealised tap density was taken as a measure of each excerpt’s tapping consensus.


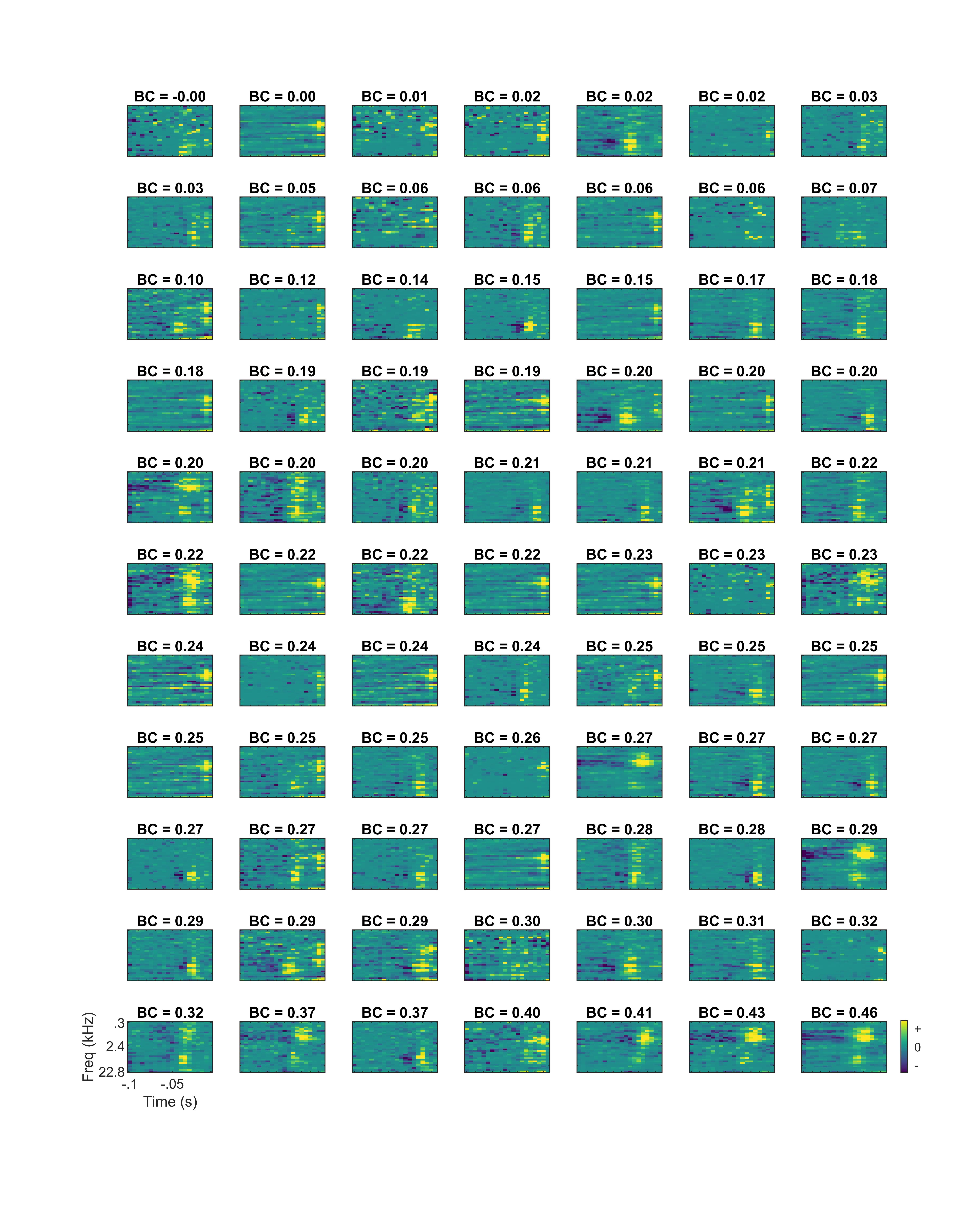


**Supplementary Fig. S7. Multiunit STRFs, ordered by increasing beat contrast.** Each panel is one multiunit’s STRF with its corresponding median beat contrast across the twenty musical excerpts. The coefficients vary greatly between multiunits and not in a systematic way with respect to beat contrast. Thus, colour scale has been set individually for each panel, but zero represents the same colour value across all panels.


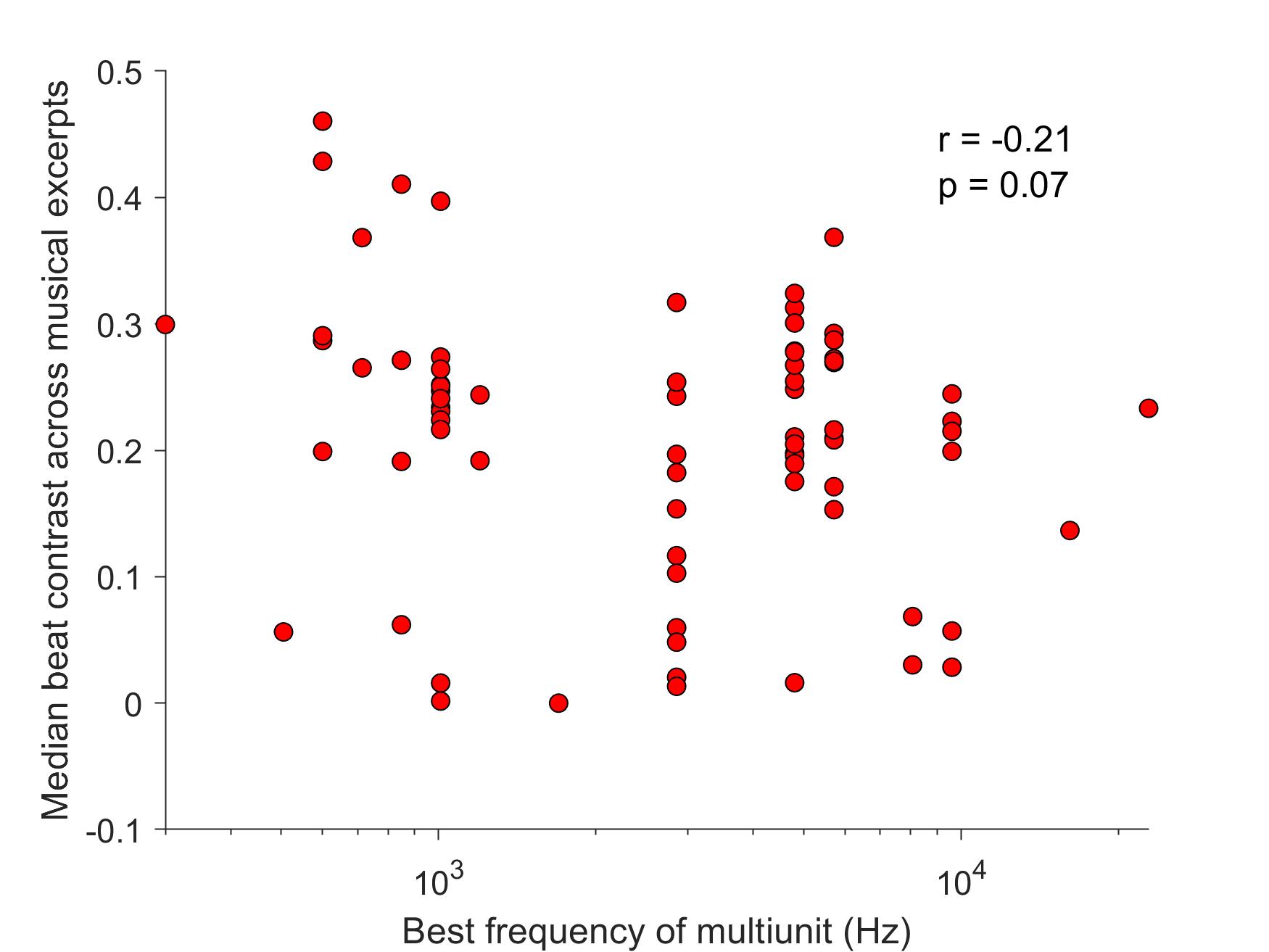


**Supplementary Fig. S8. Multiunit best frequency versus beat contrast.** Each dot is one multiunit, x-axis is the best frequency of that multiunit based on its STRF, and y-axis is that multiunit’s median beat contrast across the 20 musical excerpts. A multiunit’s best frequency did not significantly correlate with its median beat contrast (p>0.05, Pearson correlation).
